# Identification and Analysis of Amygdalin, Neoamygdalin and Amygdalin Amide in Different Processed Bitter Almonds by HPLC-ESI-MS/MS and HPLC-DAD

**DOI:** 10.3390/molecules22091425

**Published:** 2017-08-30

**Authors:** Shuya Xu, Xinfang Xu, Shaoxiong Yuan, Huan Liu, Mengnan Liu, Ying Zhang, Hui Zhang, Yan Gao, Ruichao Lin, Xiangri Li

**Affiliations:** 1 School of Chinese Materia Medica, Beijing University of Chinese Medicine, Beijing 100102, China; xushuya@bucm.edu.cn (S.X.); xuxinfang007@163.com (X.X.); 20150931937@bucm.edu.cn (H.L.); 20160931810@bucm.edu.cn (M.L.); zhang0312ying@163.com (Y.Z.); zh19930503@sina.com (H.Z.); 20150931752@bucm.edu.cn (Y.G.); linrch307@sina.com (R.L.); 2 Marketing Department, Leading Pharm Medical Technology, Inc., Beijing 100083, China; shaoxiongyuan@126.com

**Keywords:** almonds, amygdalin, neoamygdalin, processing, HPLC-ESI-MS/MS

## Abstract

Processing is a traditional pharmacy technology based on traditional Chinese medicine theory. The traditional Chinese medicine (TCM) ingredients should be processed before being used as a medicine. Processed bitter almonds are widely used in the clinic in TCM for the treatment of cough and asthma. In this work the amygdalin profile of three producing areas in China was determined, with respect to three differently processed bitter almond products: raw, stir-fried and scalded. Identification of the compounds was done by using high performance liquid chromatography coupled to electrospray ionization mass spectrometry (HPLC-ESI-MS/MS). Results indicated that amygdalin, neoamygdalin and amygdalin amide were identified in the different processed bitter almonds. Meanwhile, amygdalin was used as a standard to calculate the quantification of amygdalin and the concentration ratio of neoamygdalin and total amygdalin by HPLC-DAD. The data suggested that composition of amygdalin isomers in bitter almonds was influenced by the processing method. It also gives a new understanding of the processing principle of bitter almonds. Moreover, the classification of different processed bitter almonds can be achieved on the basis of amygdalin isomers levels.

## 1. Introduction

Processing of Chinese Materia Medica (CMM) is a pharmaceutical technique used to fulfill the different requirements of therapy, dispensing and making preparations according to traditional Chinese medicine (TCM) theory. The aims of processing are to enhance the efficacy and/or reduce the toxicity of crude drugs [1]. Almonds have high value as medicinal materials in a wide variety of foods and medicines which include sweet and bitter varieties. Almonds can maintain a healthy cholesterol level and almonds are likely to benefit other modifiable cardiovascular and diabetes risks [2]. As an antitussive medicine, the usage of almonds has a long history in China.

Amygdalin is a cyanogenic diglucoside found in bitter almonds (Semen Armeniacae Amarum) and has the effect of relieving coughs. The amygdalin can be hydrolyzed into hydrogen cyanide. This stepwise process involves the hydrolysis of amygdalin into prunasin and glucose by the action of β-glucosidase amygdalin hydrolase. When prunasin is degraded, mandelonitrile and glucose are released. The prunasin is hydrolyzed into mandelonitrile by prunasin hydrolase and is finally converted into hydrogen cyanide and benzaldehyde via β-mandelonitrile lyase [3,4,5]. In addition, some recycling pathways in almonds avoid the release of HCN and recover the reduced nitrogen and carbon from the nitrile group as ammonia and carbon dioxide [6]. Many modern studies have confirmed that a small amount of hydrogen cyanide can help to relieve cough and asthma. Additionally, benzaldehyde and hydrogen cyanide are beneficial for the patients with cancer [7], whereas at high dose they can cause dizziness, heart palpitations, headache, nausea and vomiting, and even lead to death [8]. Therefore, the quality of bitter almonds should be determined by the content of the cyanogenic glycoside amygdalin.

Under the guidance of traditional Chinese medicine (TCM) theory, bitter almonds should be processed before being used as a medicine. The change of the properties of CMM is a core tenet of the processing principle of Chinese medicine. In TCM theory, the processing principle of bitter almonds is *shameibaogan*, which means inhibiting enzymes and preserving the amygdalin in the bitter almonds. After processing, the amygdalin level in bitter almonds increases and is better more preserved. Scalded and stir-fried samples are the common clinical drugs in TCM [1,9]. The efficacy of relieving cough and asthma is enhanced in processed products. In scalded products toxicity of bitter almonds is reduced, whereas the stir-fried products are more suitable for treating long-term cough with asthma. In recent research, two isomers of amygdalin were found to exist in almonds. It is reported that d-amygdalin in aqueous solution is racemized to neoamygdalin, making it ineffective against cancer [10]. Hwang established simple methods for HPLC analysis of d-amygdalin and neoamygdalin [11]. Kang introduced a micellar electrokinetic chromatography (MEKC) method for the identification of d-amygdalin and l-amygdalin in apricot kernel extract without any pre-treatment [12]. Besides, a fast reliable method was developed by using a chiral stationary phase and HPLC [13]. However, there is no study reporting the forms of existence and the concentration ratio of amygdalin isomers in raw, scalded and stir-fried bitter almonds.

Considering of all of the above, determining the amygdalin isomers of different processed bitter almonds and discussing the processing principle of bitter almonds remains an interesting task, particularly for finding differences among the raw, scalded and stir-fried samples. In addition, we wanted to investigate the process principle of bitter almonds by modern analytical methods. To this end, the HPLC-ESI-MS/MS was used to characterize the forms of amygdalin in different processed bitter almonds from three producing area in China. Moreover, the contents of amygdalin and the relative quantities of neoamygdalin and total amygdalin were determined by HPLC.

## 2. Results and Discussions

### 2.1. Characterisation of Amygdalin Isomers

**Figure 1:**
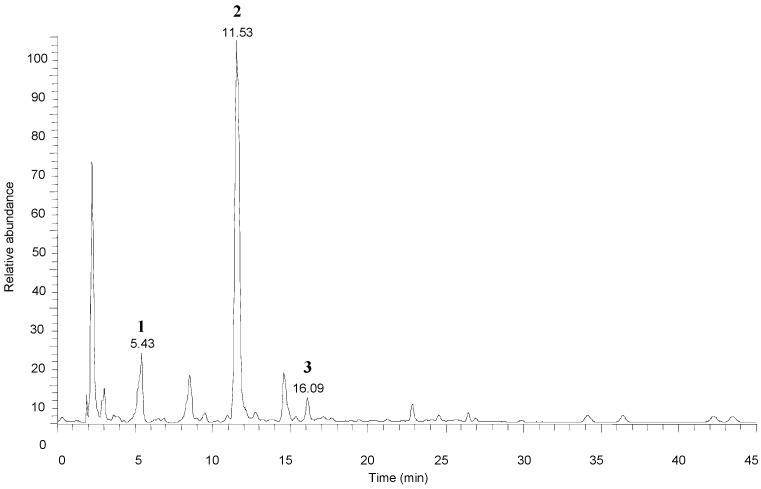
Total ion chromatogram of amygdalins characterized in bitter almonds by HPLC-ESI-MS/MS. (**1**) amygdalin amide; (**2**) amygdalin; (**3**) neoamygdalin.

**Scheme 1:**
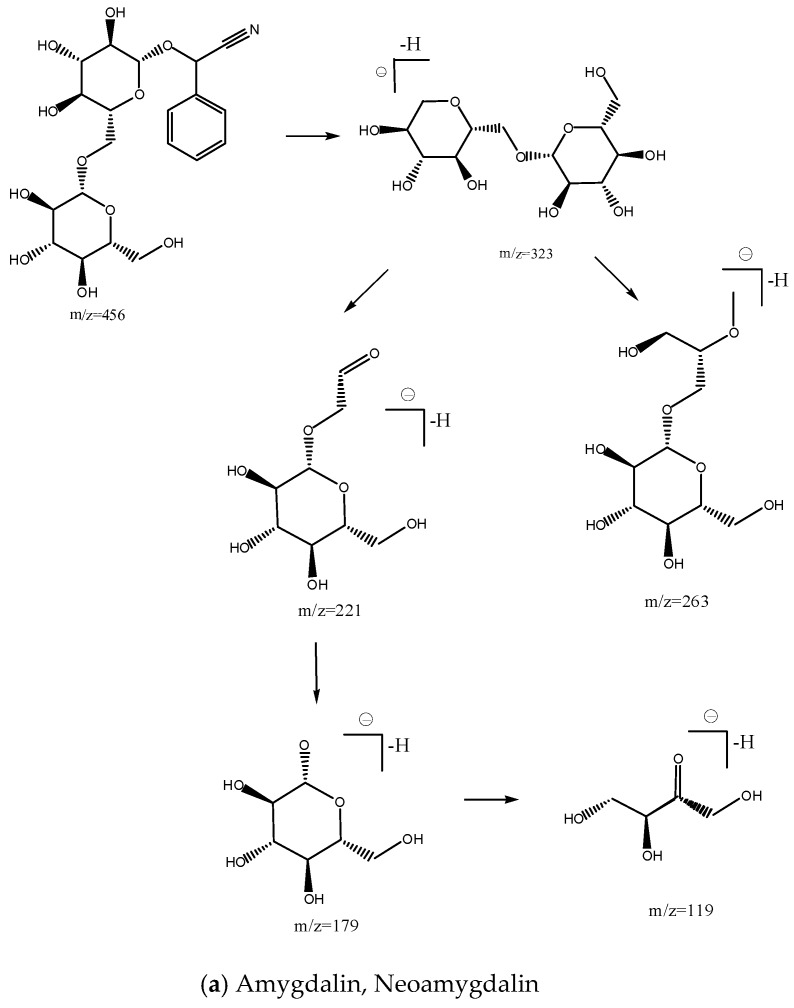

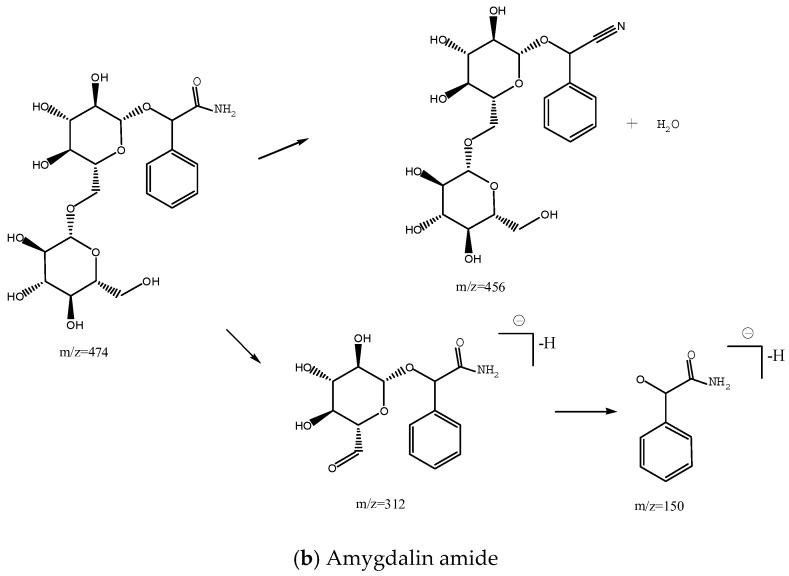
Proposed fragmentation pathway for amygdalin, neoamygdalin (**a**) and amygdalin amide (**b**) in ESI negative mode MS.

Figure 1 shows the total ion chromatogram of bitter almonds. Peaks 2 and 3 had the same molecular ion at *m*/*z* 456.15, and three mass fragments at *m*/*z* 323.09, 178.96 and 118.95 (supporting Supplementary Materials, Figures S2 and S3). The fragment at *m*/*z* 323.04 resulted from the neutral loss of the disaccharide ([M − H]^−^ − 133). Glycosidic bond cleavage and the loss of the A1 glucose resulted in the formation of fragment ions at *m*/*z* 456.15→178.96. The other peak, at *m*/*z* 118.95, resulted from the cross-ring bond cleavage of the A1 glucose. Product ions of *m*/*z* 263.06 and 221.01 were also monitored at nearly equal intensities as *m*/*z* 179. These fragment ions were also observed at *m*/*z* 456.15→263.06 and *m*/*z* 456.15→221.017 corresponding to the cross-ring bond cleavage of glucose 2 (Scheme 1) [14]. Peak 2 was identified as amygdalin, and its retention time was identical to amygdalin standard. Thus, Peak 3 could be neoamygdalin, the isomer of amygdalin. Peak 1 had a molecular ion at *m*/*z* 474.1610 and the fragments *m*/*z* 456.13, 323.08, 221.06, were same as amygdalin (Figure S1). The loss of H_2_O resulted in the formation of fragment ions at *m*/*z* 474.16→456.13. Two mass fragments were at *m*/*z* 312.11 and 150.05, both resulting from the neutral loss of the glucose (Scheme 1). Peak 1 was identified as amygdalin amide. The identities and detection characteristics (rt and mass data) of the amygdalin amide, amygdalin and neoamygdalin in different processed bitter almonds by chromatography are listed in Table 1.

Amygdalin, neoamygdalin and amygdalin amide were observed to have the same fragmentation pathways and fragment ions in the raw, scalded and stir-fried samples. It is indicated that amygdalin, neoamygdalin, amygdalin amide might exist naturally in bitter almonds.

### 2.2. Validation of HPLC Method

The HPLC method was validated according to linearity, reproducibility, limit of detection (LOD) and precision. The linearity was verified by using the standard solutions of d-amygdalin. The concentration (X) versus the peak area (Y) was plotted, and its regression line was used for the determination of sample concentrations. As a consequence, the linearity for amygdalin was linear over the concentration range 0.01–1.03 mg/mL. The regression curve was given by Y = 525036X + 5853.9, and the good linearity (correlation coefficient r = 0.9998) between Y (peak area) and X (concentration of the amygdalin) was achieved in the tested range (Figure S4). Furthermore, the LOD of amygdalin was obtained by injecting 10 µL of graded dilutions of a standard solution, followed by the comparison of peak height with baseline noise level and a signal-to-noise ratio (S/N) of 3 indicated the detection limit. The limit of detection for amygdalin was 2 µg/mL, indicating that the sensitivity of the method was satisfactory. Besides, method precision was also determined by measuring repeatability (intra-day variability) and intermediate precision (inter-day variability) of retention time and peak area for amygdalin. The relative standard deviations (RSDs) of the retention time and the peak area for amygdalin were also obtained by calculating the results on the standard solutions six times. The results showed that the intra-day reproducibility were less than 0.13% for the migration time and 0.75% for the peak areas (A), and the inter-day variability were less than 0.56% for the migration time and 0.80% for the peak areas (A), indicating that the method precision was satisfactory.

### 2.3. Analysis of Amygdalin Isomers

**Figure 2:**
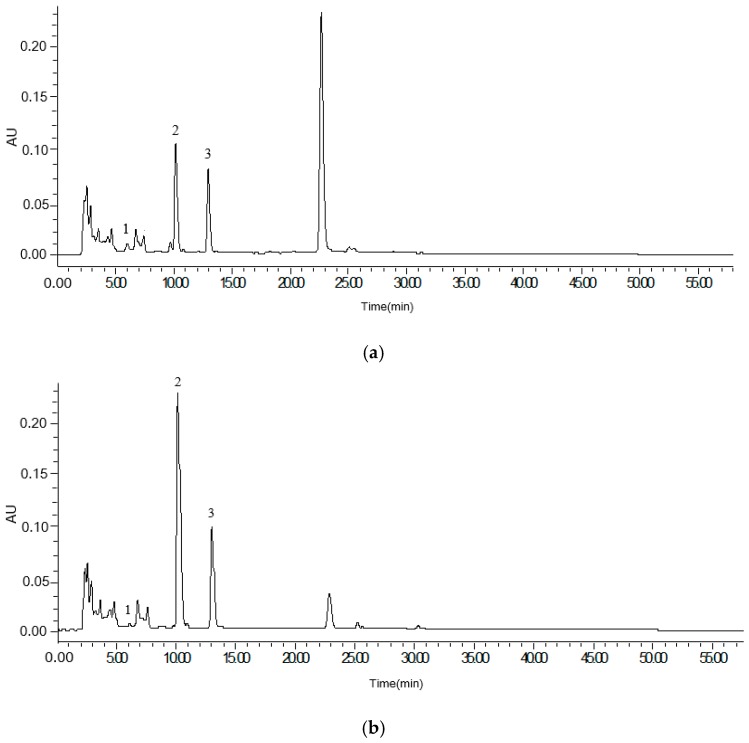

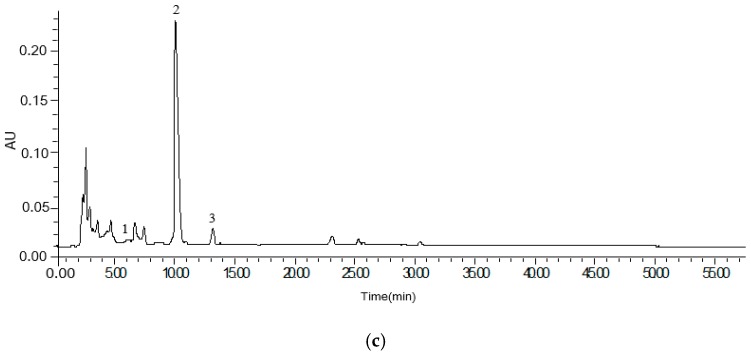
HPLC-UV chromatograms of raw bitter almond (**a**), scalded bitter almond (**b**) and stir-fried bitter almond (**c**). (**1**) amygdalin amide; (**2**) amygdalin; (**3**) neoamygdalin.

Analysis of amygdalin was achieved using a high performance liquid chromatography (HPLC) system. One of the purposes of this study was investigating the mutual transformation of the isomer of amygdalin after processing. In order to evaluate the changes between amygdalin and neoamygdalin, the contents of amygdalin and the relative quantity of neoamygdalin were determined by peak areas. All forms of amygdalin could be analysed by use of amygdalin as the standard. Figure 2 shows the HPLC-UV chromatograms of raw bitter almond (a), scalded bitter almond (b) and stir-fried bitter almond (c). Amygdalin and neoamygdalin concentrations were analysed in different processed samples (Table 2).

The mean percentage contents of amygdalin in raw, scalded, stir-fried bitter almonds were 4.02%, 5.63%, 7.56% respectively. The mean relative percentage contents of neoamygdalin in raw, scalded, stir-fried samples were 1.95%, 1.63%, 0.57% respectively. The mean relative percentages of total amygdalin in raw, scalded, stir-fried samples were 6.43%, 7.55%, 8.37% respectively.

**Figure 3:**
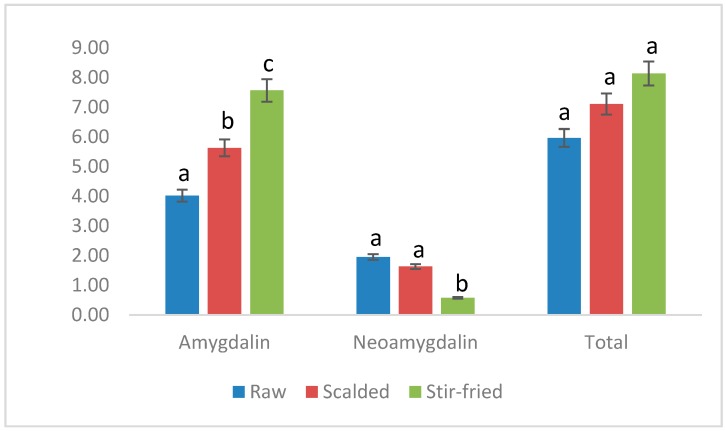
Comparison between various processed bitter almonds on different compounds. Different letters (a, b, c) within each bar of the same compound mean a statistical difference at *p* < 0.05.

The relative contents of different forms of amygdalin of various processed bitter almonds were used to evaluate the changes in almonds after processing (Figure 3). More concretely, the contents of amygdalin in processed products were significantly higher than in the raw almonds (*p* < 0.01). Amygdalin values were found to be notably higher in stir-fried varieties than in scalded samples (*p* < 0.05). The lowest neoamygdalin relative concentration was found in stir-fried varieties. However, there was no significant difference among the three processed products in the relative contents of total amygdalin (*p* > 0.05). The peak area of amygdalin amide was decreased after heating, which means the amygdalin amide may dehydrated and changed to amygdalin. The results indicated that the composition of amygdalin in almonds is largely influenced by processing method. It proved that heating inhibited the enzyme that decomposes amygdalin, and at the same time it made neoamygdalin and amygdalin amide change into amygdalin.

### 2.4. Principal Component Analysis

**Figure 4:**
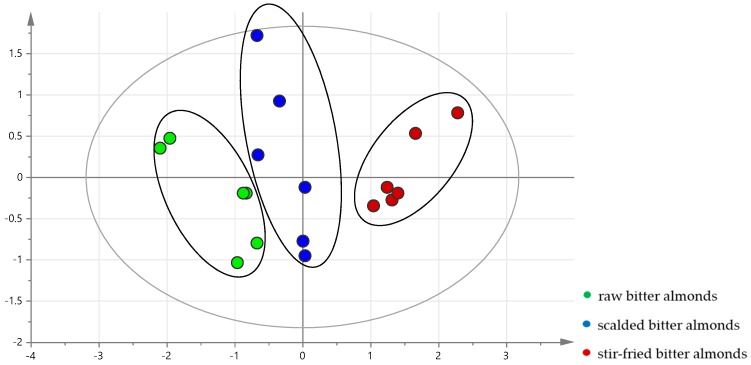
PCA score plots of different processed bitter almonds.

There was no change in the composition of the three kinds of processed products. However, the concentration ratios of amygdalin, neoamygdalin were different after processing. Amygdalin content and the relative content of neoamygdalin were used as variables for PCA. The HPLC data were processed by unsupervised PCA [15]. After Pareto (Par) Scaling with mean-centering, the data were displayed as score plots (Figure 4). It is evident from Figure 4 that most raw, scalded and stir-fried bitter almonds were clearly clustered into three groups, indicating there were differences among three processed samples. Raw bitter almonds were farther from stir-fried bitter almonds, while scalded bitter almonds were between the other two processed products. The result showed that the transformation between amygdalin and neoamygdalin were carried out step by step in raw, scalded and stir-fried bitter almonds.

## 3. Materials and Methods

### 3.1. Chemicals

Amygdalin (>99%) was obtained from the Chinese Food and Drug Inspection Institute (Beijing, China). HPLC grade methanol and formic acid were purchased from Fisher Scientific (Fair Lawn, NJ, USA). Ultrapure water was filtered through a Milli-Q plus system (Millipore, Bedford, MA, USA). All other chemicals were of analytical reagent grade.

### 3.2. Almond Samples

Six batches of bitter almonds were collected at Hebei Province, Shanxi Province, and the Northeast of China. The collection occurred during 2013 and 2014. The samples were identified as *Prunus armeniaca* L. by Professor Xiangri Li from the Institute of Traditional Chinese Medicine, Beijing University of Chinese Medicine. All Samples were preserved at the Beijing University of Chinese Medicine.

### 3.3. Sample Processing, Extraction

Removal of foreign matters from crude almonds gave the raw almonds. Briefly, raw almonds were blanched in boiling water for about 10 min and then the almonds were taken out to remove the skins (seed coats) by hand to give the scalded almonds. The scalded samples were next placed in a pot, heated with constant tossing or stirring until scalded almonds turned yellow, and thus the stir-fried samples were obtained.

To determine the amygdalin in the bitter almonds, the raw, scalded and stir-fried samples were analyzed. The powdered seeds of raw, scalded and stir-fried almonds (2 g) were extracted with 100 mL of 70% aqueous methanol under ultrasonic extraction conditions over 30 min at room temperature. The 70% methanolic extract was retained and filtered. The residue was extracted with 100 mL of 70% aqueous methanol at ultrasonic by a two-step and repeated the above operation. The combined solution was concentrated to dryness under reduced pressure. The residue was dissolved in methanol in 10 mL volumetric flask for HPLC-DAD analysis. The solution diluted five times was analyzed by HPLC-ESI-MS/MS.

### 3.4. Preparation of Standard Solution

Commercially available standards of d-amygdalin (5 mg) was dissolved in 70% MeOH/H_2_O solution (*v*/*v*) (5 mL), and used as standard stock solutions for generating calibration curves. Working standard solutions were further obtained by appropriate dilution of the stock standard solutions with 70% MeOH/H_2_O solution (*v*/*v*). The sample solutions were filtered through a 0.22 µm syringe filter and were degassed using an ultrasonic bath for 2 min prior to use. The standard solutions was injected to generate a five point calibration curve for amygdalin. All the solutions prepared were stored in the dark at 4 °C until being used.

### 3.5. HPLC Equipment and Conditions

The analysis of amygdalin isomers was carried out on a an Agilent 1100 high performance liquid chromatography system (Agilent Technologies, Palo Alto, CA, USA), equipped with a diode array detection detector, model 1100 pumps, an auto sampler and a thermostat was controlled by Agilent 1100 Chemstation. Separation of compounds was achieved using an Eclipse EXTEND C_18_ column (4.6 mm × 250 mm, 5 µm) kept at 35 °C. A linear gradient consisting of solvent A (methanol) and solvent B (0.1% formic acid in water) was applied as a flow rate of 1 mL/min as follows: 15–33% A, 0–20 min; 33–40% A, 20–25 min; 40–45% A, 25–45 min. The column was re-equilibrated between injections for 10 min with initial mobile phase. The injection volume was 20 µL, and the detection wavelength is 254 nm. HPLC solvents had to be prepared daily in order to prevent the modification of the compounds in retention time.

### 3.6. HPLC-ESI-MS/MS Equipment and Conditions

The HPLC system employed an electrospray ionization (ESI) to a LTQ-Orbitrap XL system (Thermo Scientific, Waltham, MA, USA). Negative ESI mode was used to verify the identities of the amygdalin in bitter almonds. Source parameters: capillary temperature 350 °C, sheath gas flow rate 30 arb, aux gas flow rate 10 arb, spray voltage 3 kV, capillary voltage-35 kV, tube lens voltage-110 V. The identification of amygdalin in bitter almond was directed through an ion trap MS fitted with an electrospray interface operating in negative ion mode scans from *m*/*z* 100 to 1200. To obtain the best selectivity, sensitivity and resolution the optimized HPLC-ESI-MS/MS method described above was applied to the identification of amygdalin compounds in bitter almonds. Extracts were diluted before injection which exceeded the linear range of the standard curve. The different structures of compounds were identified based on a complete match of their HPLC retention time (rt), *m*/*z* of their molecular ions, and MS fragmentation patterns.

### 3.7. Statistical Analysis

Results for relative contents are expressed as the mean of three measurements ± standard deviation (SD).The statistical analyses were performed using SPSS statistics software (v. 17.0, SPSS, Inc., Chicago, IL, USA). Significant differences of three forms of amygdalin relative concentrations among various processed bitter almonds were determined using one-way ANOVA followed by the Duncan’s multiple-range test at *p* < 0.05. Multivariate statistical analysis of HPLC data was carried out using SIMCA software 13.0 (Umetrics, Umeå, Sweden). Principal component analysis (PCA) was carried out to compare the holistic quality and to find characteristic components of raw, scalded and stir-fried bitter almonds.

## 4. Conclusions

In summary, a HPLC-ESI-MS/MS method was developed to identify the characteristics of amygdalin isomers in bitter almonds. For the first time, the presence of amygdalin, neoamygdalin and amygdalin amide in different processed bitter almonds were reported. The identification of compounds were based on their exact mass, characteristic fragmentation pathways and retention times.

Furthermore, the quantification of amygdalin was achieved by HPLC-DAD. The amygdalin was used as a standard to calculate the relative contents of neoamygdalin and total amygdalin, revealing the transformation of amygdalin isomers in different processed bitter almonds. In this study, there was a mutual transformation between amygdalin and neoamygdalin during processing. Meanwhile, the amygdalin amide may be dehydrated and changed to amygdalin during the processing. There were significant differences in the contents of amygdalin among the three processed products. The stir-fried samples had statistically higher amygdalin concentrations than raw and scalded samples. Alternatively, the relative contents of neoamygdalin in stir-fried and scalded samples were lower than in raw bitter almonds. There was no significant difference among the three processed products in the relative contents of total amygdalin isomers. It means that neoamygdalin may transform into amygdalin during the processing. This provides a new understanding of the processing principle of bitter almonds. Besides the inhibiting enzymes and preserving the amygdalin, the neoamygdalin and amygdalin amide may change into amygdalin, thus increasing the content of amygdalin.

Meanwhile, one of the main points of this study was the result of PCA as discrimination among raw bitter almonds, stir-fried samples and scalded samples. The results showed that the raw bitter almonds and its processed products can be clearly distinguished and classified. These data can lead to a new understanding of the mechanisms of processing and a brand new outcome in human studies about amygdalin isomers.

## Figures and Tables

**Table 1:** Compounds in different processed bitter almonds identified by HPLC-ESI-MS/MS.

Sample	Rt (min)	Compound	Mol. Ion [M − H]^–^	Primary Fragment
raw bitter almond	5.43	amygdalin amide	474.1610	312.1082, 150.0560, 323.0784, 263.0289, 221.0626
	11.53	d-amygdalin	456.1501	323.0973, 263.0766, 221.0899, 179.0197
	16.09	l-amygdalin	456.1532	323.0808, 262.8893, 221.0302, 178.9521
scalded bitter almond	5.21	amygdalin amide	474.1609	312.1082, 150.0560, 323.0616, 263.0547, 221.0994
	11.01	d-amygdalin	456.1501	323.0974, 263.0765, 221.0201, 178.9646
	16.09	l-amygdalin	456.1508	323.1771, 263.1088, 221.01, 178.86
stir-fried bitter almond	5.36	amygdalin amide	474.1610	312.1082, 150.0560, 323.1720, 263.0850, 221.0170, 178.9606
	12.03	amygdalin	456.1501	323.0973, 263.0766, 221.0641, 179.0558
	15.97	neoamygdalin	456.1508	323.0952, 263.0011, 221.0641, 178.9624

**Table 2:** The percentage of amygdalin and the relative contents of other forms in raw, scalded, and stir-fried bitter almonds (mean ± SD, n = 3).

Origin	Sample	Amygdalin (%)	Neoamygdalin (%)	Total (%)
Shanxi	raw	4.51 ± 0.04	1.76 ± 0.02	6.27 ± 0.01
	scalded	5.45 ± 0.26	1.60 ± 0.43	7.04 ± 0.18
	stir-fried	7.06 ± 0.14	0.47 ± 0.01	7.54 ± 0.14
Hebei	raw	3.70 ± 0.43	1.32 ± 0.02	5.02 ± 0.32
	scalded	4.75 ± 0.13	0.88 ± 0.09	5.18 ± 0.31
	stir-fried	6.80 ± 0.35	0.60 ± 0.01	7.39 ± 0.38
the Northeast	raw	3.84 ± 0.22	2.74 ± 0.05	6.61 ± 0.20
	scalded	6.69 ± 0.40	2.42 ± 0.45	9.09 ± 0.78
	stir-fried	8.82 ± 0.72	0.64 ± 0.15	9.46 ± 0.56

## Supplementary Materials

Supplementary materials are available online. Figure S1: Negative mode product ion spectrum of amygdalin amide. a. Spectrum of MS, b. Spectrum of MS^2^, Figure S2: Negative mode product ion spectrum of amygdalin. a. Spectrum of MS, b. Spectrum of MS^2^, c. Spectrum of MS^3^, Figure S3: Negative mode product ion spectrum of neoamygdalin. a. Spectrum of MS, b. Spectrum of MS^2^, c. Spectrum of MS^3^, Figure S4: Calibration curves of d-amygdalin.
