# Supplementary material for: Identification and Analysis of Amygdalin, Neoamygdalin and Amygdalin Amide in Different Processed Bitter Almonds by HPLC-ESI-MS/MS and HPLC-DAD
Source: Molecules. 2017 Aug 30;22(9):1425. doi: 10.3390/molecules22091425 (PMC6151405; doi:10.3390/molecules22091425)
Supplement: Supplementary file 1 [file molecules-22-01425-s001.pdf]

(a)

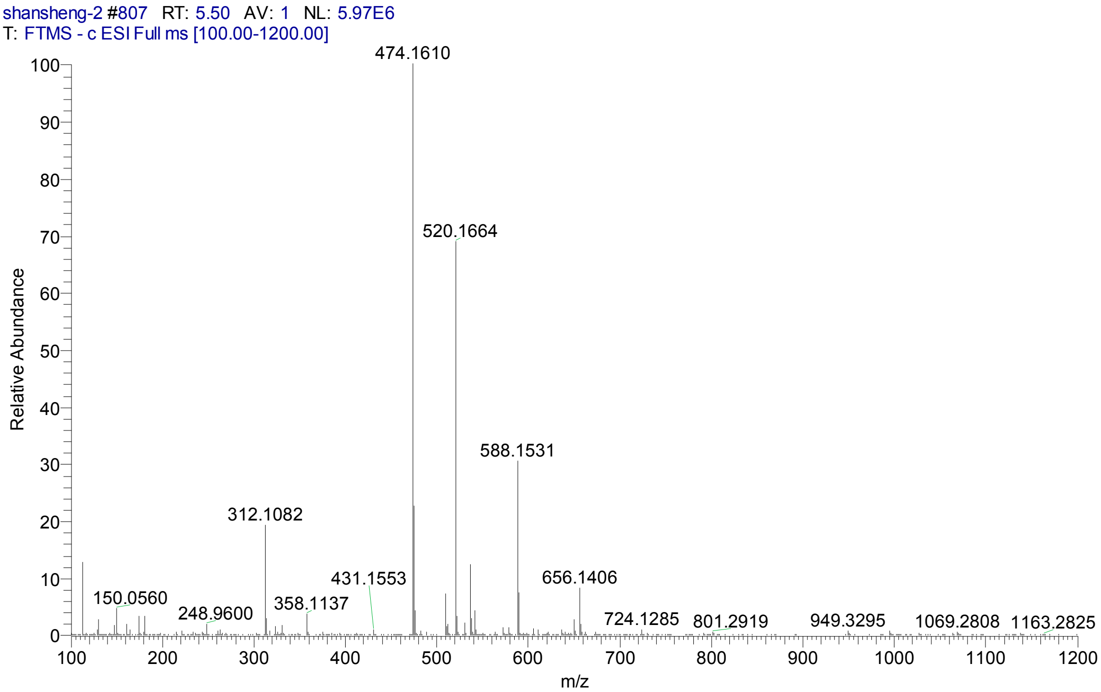

(b)

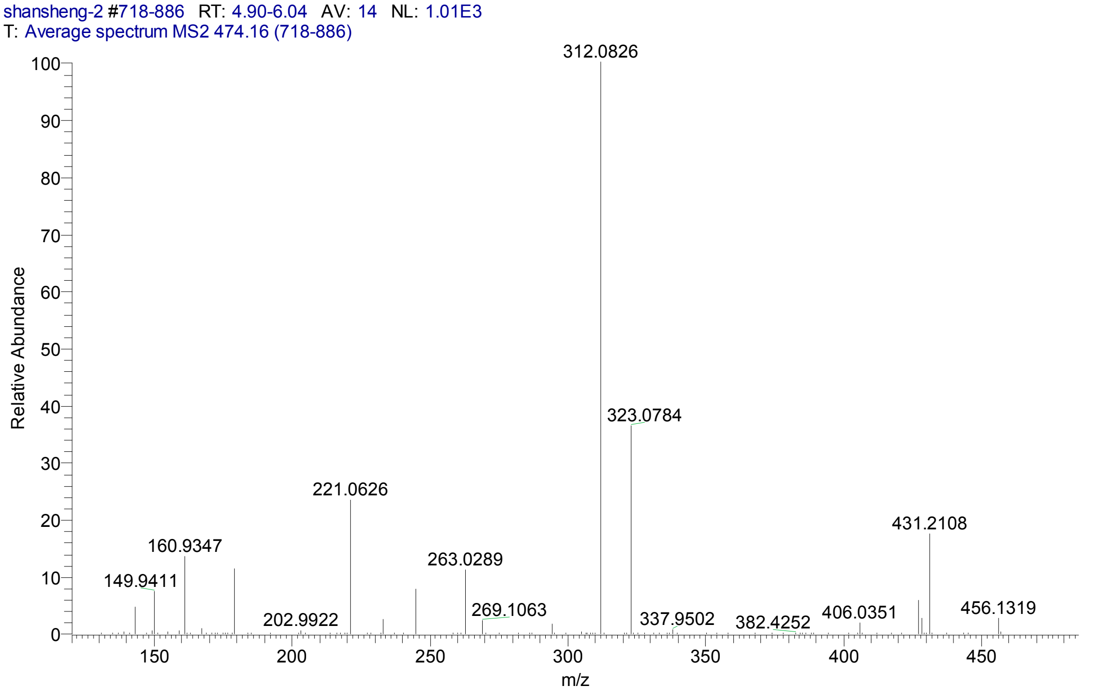

Figure 1. Negative mode product ion spectrum of amygdalin amide. a. Spectrum of MS, b. Spectrum of MS^2

(a)

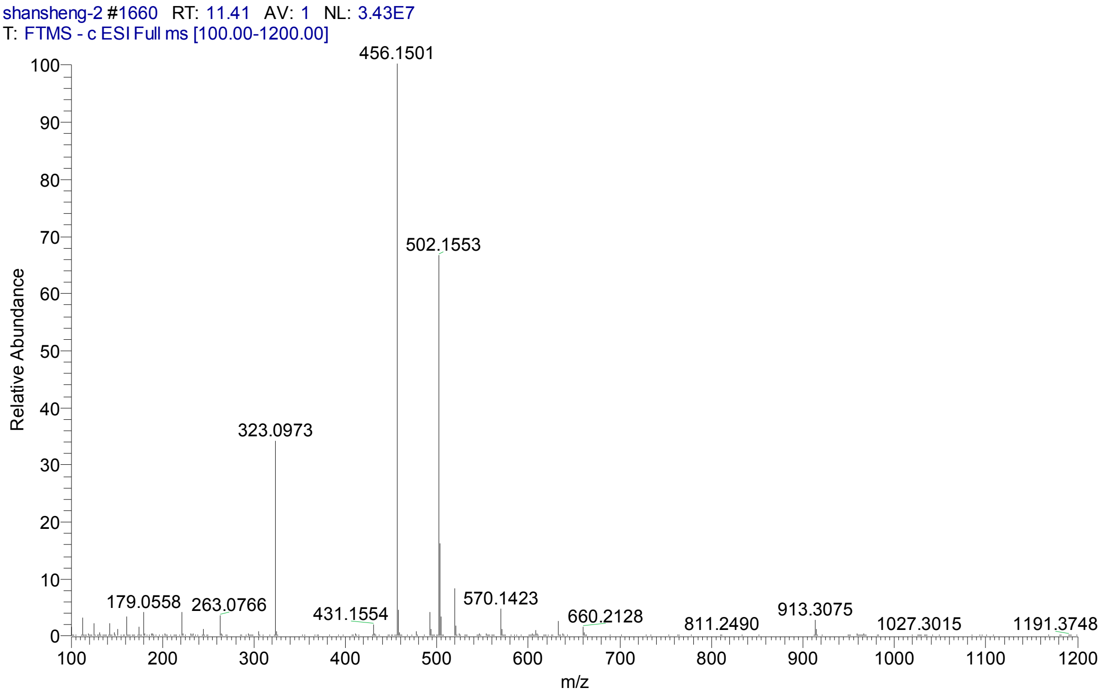
shansheng-2 #1660 RT: 11.41 AV: 1 NL: 3.43E7
T: FTMS - c ESI Full ms [100.00-1200.00]
456.1501
502.1553
323.0973
179.0558
263.0766
431.1554
570.1423
660.2128
811.2490
913.3075
1027.3015
1191.3748
Relative Abundance
m/z

(b)

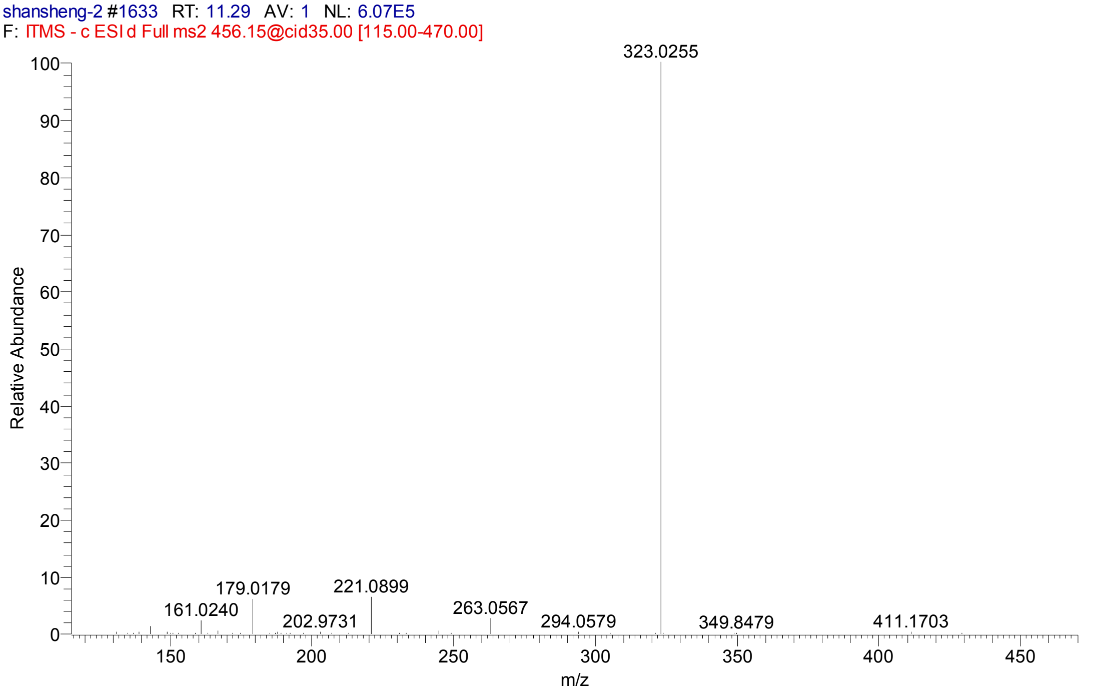
shansheng-2 #1633 RT: 11.29 AV: 1 NL: 6.07E5
F: ITMS - c ESI d Full ms2 456.15@cid35.00 [115.00-470.00]
323.0255
179.0179
221.0899
161.0240
202.9731
263.0567
294.0579
349.8479
411.1703
Relative Abundance
m/z

(c)

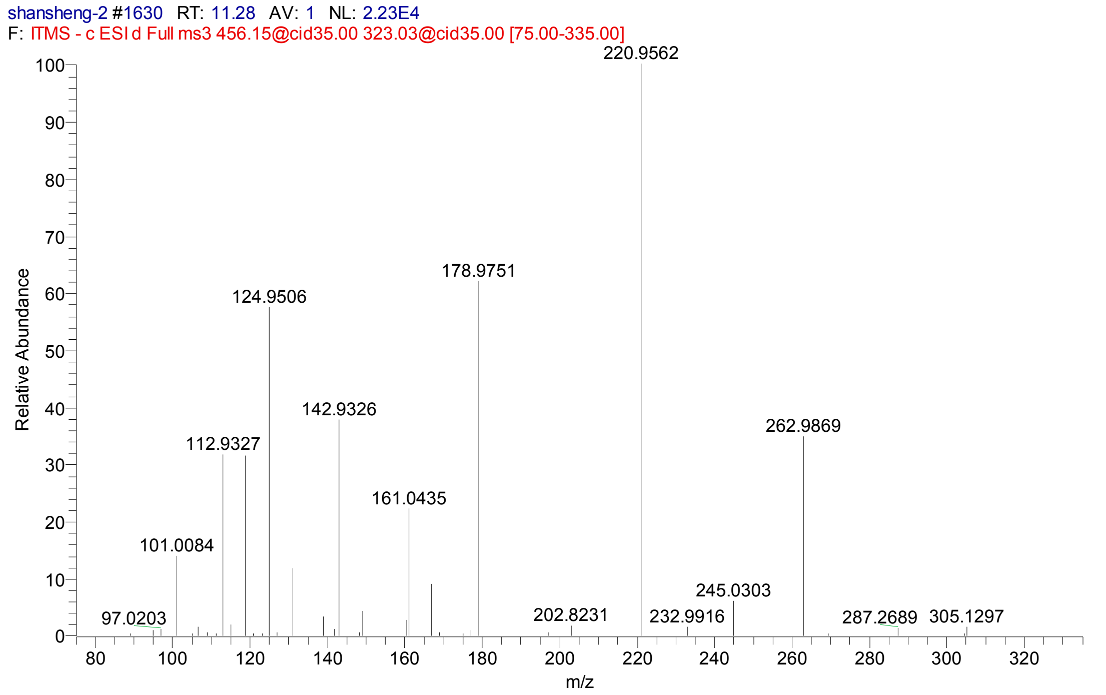

Figure 2. Negative mode product ion spectrum of amygdalin. a. Spectrum of MS, b. Spectrum of MS^2, c. Spectrum of MS^3

(a)

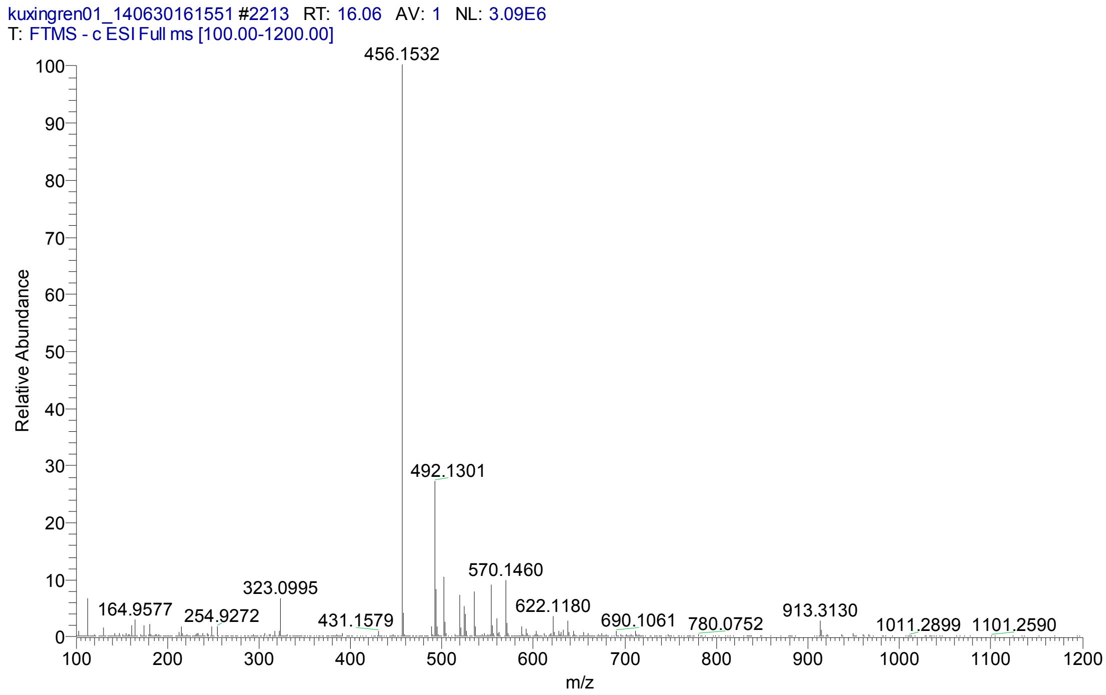

(b)

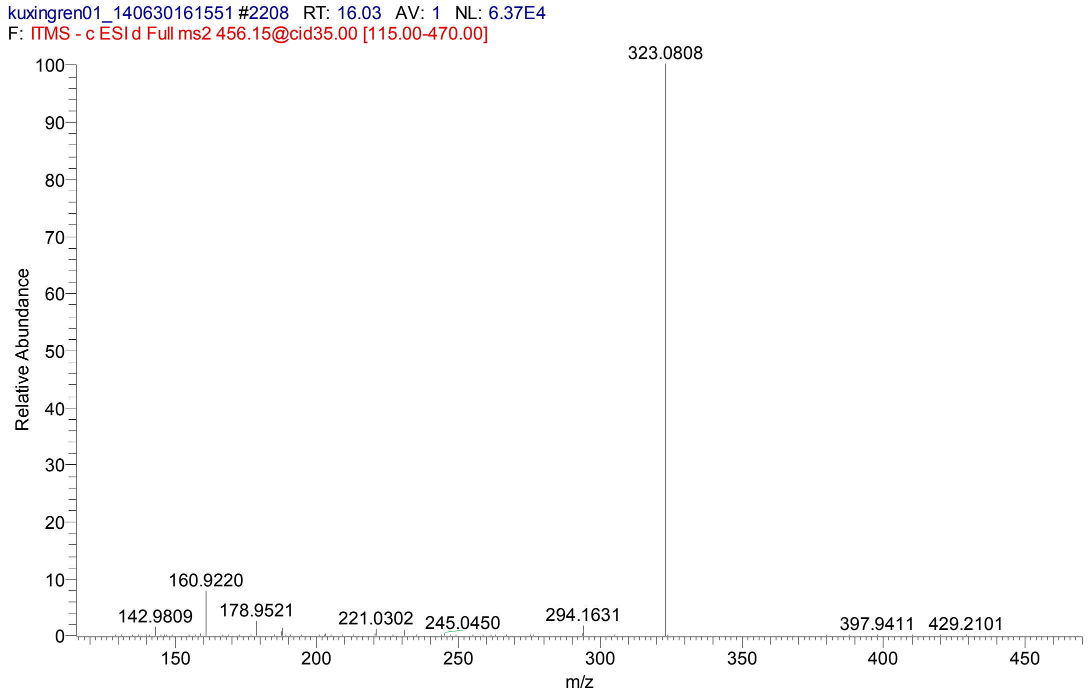

(c)

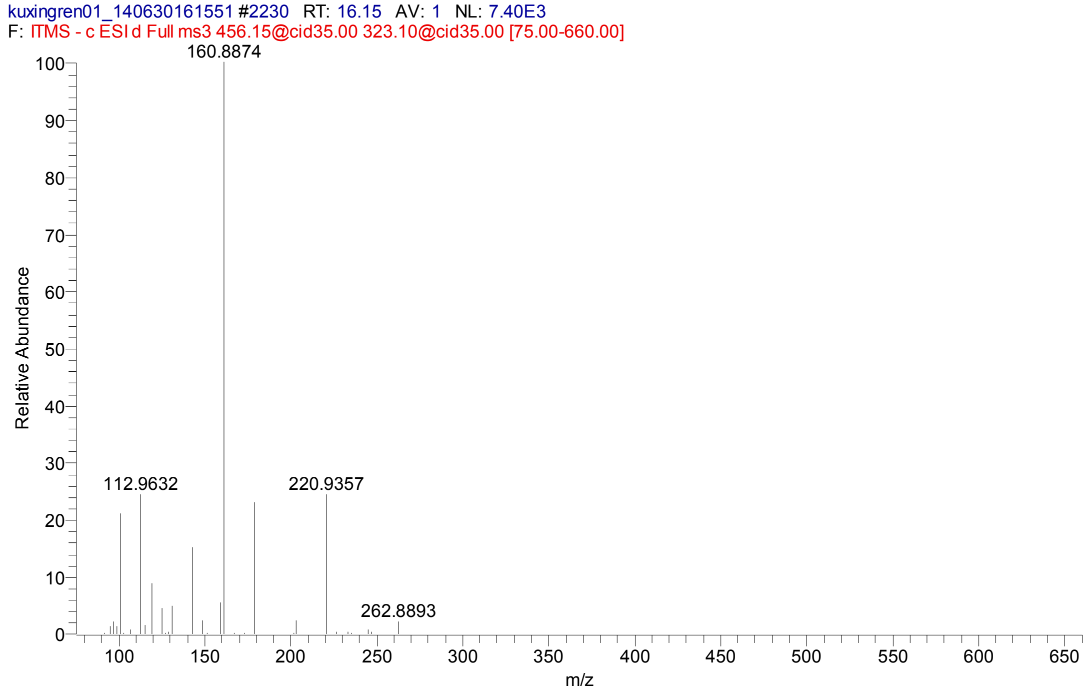

Figure 3. Negative mode product ion spectrum of neoamygdalin. a. Spectrum of MS, b. Spectrum of MS^2, c. Spectrum of MS^3

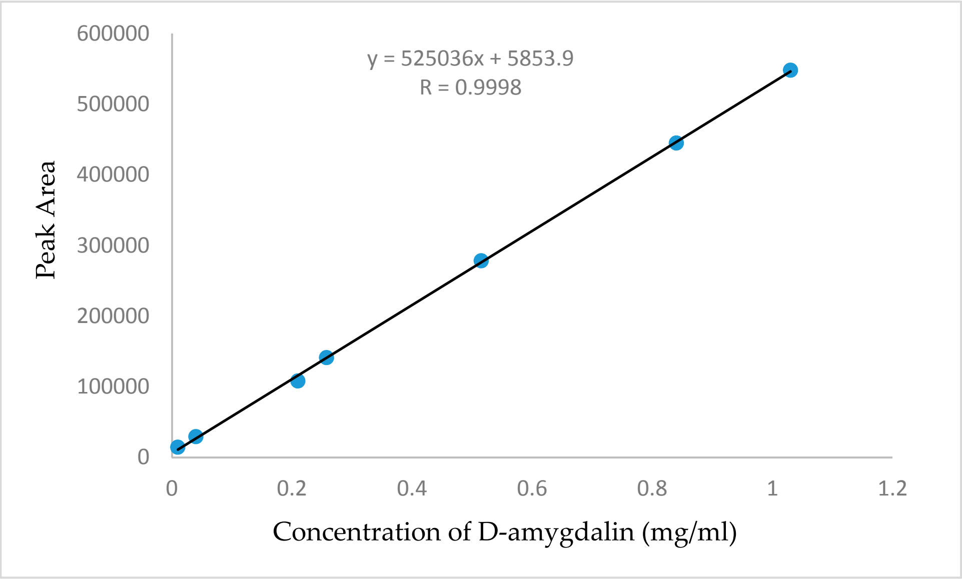

Figure 4. Calibration curves of D-amygdalin
